# Special Issue: “Management of Early Stage Cervical Cancer”

**DOI:** 10.3390/cancers15082343

**Published:** 2023-04-18

**Authors:** Camilla Certelli, Luigi Pedone Anchora, Valerio Gallotta

**Affiliations:** 1 Gynecologic Oncology Unit, Department of Woman, Child and Public Health, Fondazione Policlinico Universitario A. Gemelli IRCCS, 00168 Rome, Italy; certelli.camilla@gmail.com; 2 Institute of Obstetrics and Gynecology, Università Cattolica del Sacro Cuore, 00168 Rome, Italy

Cervical carcinoma is a common gynecological malignancy that remains a challenge for oncologic gynecologists around the world. Despite prevention, it still causes morbidity. The early stages are highly curable and the standard treatment is represented by surgery. However, the management of this tumor has changed over the years.

This Special Issue, which comprises 10 papers (8 original articles and 2 reviews), addresses various aspects concerning the state of the art and future perspectives in the field of early-stage cervical cancer.

The review of the literature by Guimarães and colleagues discussed the different aspects of this cancer (FIGO staging system update, sentinel lymph node mapping, surgical approach, conservative management, and fertility preservation) with the aim of developing a more tailored treatment to prevent morbidity and assure oncologic safety [1].

The FIGO 2018 staging system update marked a major change as, from that moment, lymph node positivity assumed an important prognostic role in the upstaging of the disease and, consequently, different patient management methods. For this reason, clinical staging is no longer the only focus, but rather a part of the efforts in the management of early-stage cervical cancer is focused on the diagnosis and preoperative evaluation of the extent of the disease and the presence of possible lymph node metastasis.

The review by Park and Kim evaluated the MRI findings in early cervical cancer, underlying how preoperative imaging may help in the modulation of surgery and in the choice of a minimally invasive approach [2]. Jeong et al., in particular, reported the diagnostic efficacy of three-tesla MRI in 342 patients with stage IB1 cervical cancer. They showed that patients with a non-visible tumor had more favorable characteristics in terms of histological features and oncological outcomes compared with patients with visible tumors, suggesting how preoperative imaging may guide surgical radicality. Interestingly, they reported a significant difference in the number of squamous cell carcinomas (SCCs) versus non-SCCs between the two groups that, on the one hand, may have affected the oncological outcomes, but on the other hand, may suggest different MRI characteristics according to the histological type [3].

The prediction of lymph node involvement is an interesting topic, especially regarding pathological evaluation. In the sentinel node era, two papers focused on the research of features to predict lymph node positivity. Bizzarri et al. analyzed the incidence of parametrial-positive lymph nodes through the ultrastaging of parametrial tissue, with a reported rate of 3.1%. They showed that the lymph node involvement of the parametrium was associated with lymph node metastasis, although the sensitivity was found to be low (16.7%), which was probably because of the low-risk group of patients included in the study (most of them had tumor diameters < 20 mm and negative LVSI) [4]. However, the positivity of parametrial lymph nodes may lead to a classification dilemma of staging, as with FIGO 2018 IIB vs. IIIC1p, or may affect the choice of adjuvant therapy.

Balaya and colleagues analyzed the risk factors that used to be associated with a higher incidence of positive lymph nodes in conization specimens: a depth stromal invasion <10 mm and no LVSI had a lower risk of micro- and macro-metastasis in SNL [5].

Considering the potential benefit in terms of the relapse and survival of patients undergoing conization, together with the diagnostic information that may enhance our ability to make the appropriate recommendation, the SUCCOR study showed that cervical conization, especially in small tumors (up to 2 cm, as conization has no role in large tumors), may be used for tailoring the surgery and the choice of surgical approach in cervical cancer patients [6]. Regarding the potential protective effect of conization, despite the publication of the LACC trial [7], the reasons for minimally invasive surgery having worse oncological outcomes have not been well established. In this context, the analyses of the SUCCOR group may suggest the hypothesis that in minimally invasive surgery the tumor experiences less exposure and manipulation due to prior diagnostic conization [8,9].

Fusegi et al. presented the results of their no-look no-touch technique (consisting of four steps to prevent tumor spillage), showing similar DFS rates when compared with the laparotomic approach, even when the tumor diameter exceeds 2 cm [10].

The most popular classifications of radical hysterectomy are based on the lateral extent of the resection (Piver or Querleu-Morrow). In his report, Muallem presented a new classification based on a three-dimensional way of tumor spreading, including the removal of the vaginal cuff and the paracolpium as an essential part of the surgical procedure [11]. Furthermore, this new classification is based on the concept that when there is a risk of parametrial/paracolpium involvement according to the local extent of the disease, these tissues should be completely removed to allow their evaluation, since their invasion is reported to be mainly discontinuous, in a way similar to lymphovascular space infiltration or lymph node metastasis [12].

Since almost 40% of all cervical diagnoses are made in women aged 20–39 years [13], the interest in strategies for fertility preservation is becoming more widespread. However, fertility-sparing management in cervical cancer patients when the tumor diameter exceeds 2 cm is considered to be an experimental approach [14,15]. Buda and colleagues revised the literature about the use of neoadjuvant chemotherapy followed by fertility-sparing surgery in women with stage IB2 cervical cancer. Although in a total of 114 patients they reported an optimal pathological response rate of 60.9% and a pregnancy rate of 85.7%, 61.1% of patients experienced miscarriages or pre-term labor, underlying the fact that there is not enough evidence in the literature to draw firm conclusions [16]. As in locally advanced tumors, the pathologic residual tumor after neoadjuvant therapy seems to be the most important prognostic factor [17,18,19].

On the other hand, the incidence of disease at a fertility age poses the problem of pregnancy-associated cervical cancer, which has increased in Japan in recent years. For this reason, Enomoto and colleagues published a multicenter survey to try to understand this topic, drawing attention to a period of poor adherence to the human papillomavirus (HPV) vaccination. They reported different treatment strategies (conization, trachelectomy and neoadjuvant treatment) with no differences in terms of oncological outcomes, as well as a surprising tendency for a longer duration of pregnancy in patients who underwent trachelectomy and a significantly higher incidence of fetuses that were small for their gestational age in the neoadjuvant group [20].

Last but not least, the research was continued in the study of the pathogenesis of cervical cancer. The most important cause of cervical carcinogenesis is represented by HPV, especially HPV16 and HPV18, accounting for approximately 50% and 20%, respectively, of the detected cases in cervical cancer [21]. From 2000, different studies showed that HOXD9 regulates the early promoter of HPV16. Hayashi et al. confirmed its role even in the regulation of HPV18, implementing the results in the research of a target therapy especially for the worse prognoses HPV-related histotypes [22].

In conclusion, this Special Issue presents updated research on topics that are useful for gynecologic oncologists in the management of early-stage cervical cancer.
